# Growth under pressure: The pros and cons of polyploidy induced by stress

**DOI:** 10.1073/pnas.2522063123

**Published:** 2026-05-26

**Authors:** Lilijana Sarabia Oliver, Paulo B. Belato, Joshua Silva, Anna Selmecki, Donald T. Fox, Adrienne H. K. Roeder

**Affiliations:** ^a^ Polyploidy Integration and Innovation Institute, Cornell University, Ithaca, NY 14853; ^b^Section of Plant Biology, School of Integrative Plant Science, and Weill Institute for Cell and Molecular Biology, Cornell University, Ithaca NY 14853; ^c^ Department of Pharmacology and Cancer Biology, Duke University School of Medicine, Durham, NC 27710; ^d^ Department of Microbiology and Immunology, University of Minnesota School of Medicine, Minneapolis, MN 55455

**Keywords:** polyploidy, endocycles, oxidative stress, genome instability, stress

## Abstract

In native environments, organisms are faced with an array of acute or chronic stresses. These stresses include toxins, pathogens, and physical injury. An increasingly recognized response to diverse stresses is whole genome doubling or polyploidy. This transformative cellular property alters genome integrity, cellular structure, and tissue architecture. Whether polyploidy is a positive, negative, or neutral outcome of a stress is a current topic of investigation in numerous contexts including during fungal infection of plants and animals, during regeneration of wounded tissues, and in human diseases such as cancer. In this review, we highlight the wide range of stresses that promote polyploidy in fungal, plant, and animal contexts. Specifically, we highlight major mechanisms that lead to stress-induced polyploidy within somatic or germline tissues through alteration of the cell cycle. We discuss the impact of such stress-induced polyploidy on genomes, cells, and tissues and emphasize commonalities across organisms and biological scales. A common theme that has emerged is that polyploidy facilitates numerous subsequent genomic and cellular changes following abrupt stresses, and these changes can impact tissue architecture and function.

Polyploidy, or whole genome duplication, is a transformative cellular process associated with increased cell size (1–3). Polyploidization can occur at the somatic or germline level, resulting in endopolyploid cells or entirely polyploid organisms, respectively (3). While polyploidy is often a normal product of many developmental processes, in recent years it has become clear that polyploidy frequently results from a variety of stress conditions (3–5). Such instances of polyploidy have previously been studied in disparate biological contexts, although common themes across diverse disciplines such as agriculture and medicine are emerging (5, 6). As a result, there is currently a significant interest in characterizing how external stresses can promote increased occurrence of polyploidy, and the resulting impacts on genomes, cells, tissues, and organisms.

In many stress-related contexts, polyploidy provides a benefit, whereas in others it may exacerbate the stress. Additionally, there are many examples of stress-induced polyploidy for which we do not know the impact. This perspective highlights distinct stresses that promote polyploidy in fungi, plants, and animals. Herein, we broadly define “stress” as an acute change in an organism’s environmental conditions, either biotic or abiotic, and including physical or chemical effects experimentally inflicted upon an organism. Our focus here is on polyploidy that arises upon stress within cells or tissues, and/or through alteration of the mitotic cell cycle. Specifically, incomplete mitotic cell cycles with continued cycles of S-phase can produce polyploid somatic cells, termed endopolyploidy if occurring in multicellular organisms (Fig. 1). Such cycles can omit cytokinesis, anaphase, or M-phase altogether (Fig. 1). For the impact of other related topics, such as stress-induced polyploidy through cell fusion or stress-induced organismal polyploidy, we refer the reader to other excellent reviews (5, 7–10).

**Fig. 1.**
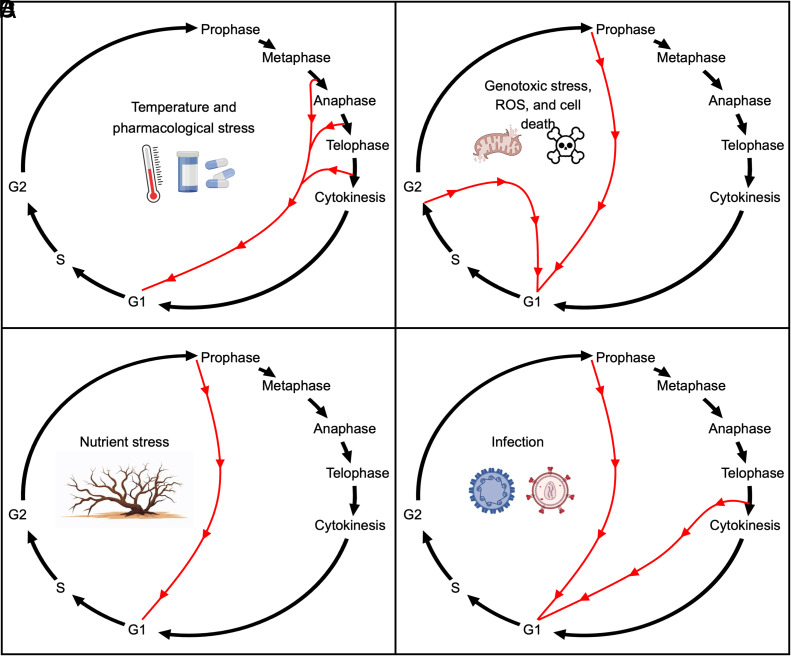
Causes of stress-induced polyploidy. Various stresses can lead to skipping of one or more phases of a mitotic cell cycle, which leads to a doubling of the ploidy. Black arrows depict mitotic cell cycle progression, while red arrows indicate distinct stress-induced cell cycle trajectories that lead to polyploidy. (*A*) Temperature or pharmacological stresses can interfere with metaphase, anaphase, or cytokinesis. In each case, this can lead to polyploidy following a subsequent S-phase. (*B*) Genotoxic stresses such as ROS lead to DNA breakage, which can arrest the cell cycle during S or G2 phases. However, bypass of this arrest through skipping of downstream cell cycle events can lead to polyploidy. Similarly, cell loss through death, combined with underlying tissue-specific gene expression can suppress the mitotic phases of the cell cycle. (*C*) Nutrient stress can promote bypass of a G2 arrest associated with skipping of downstream cell cycle events, causing subsequent polyploidy. (*D*) Infection stress can promote cell cycle reentry from G0, and such cycling cells often skip mitosis to become polyploid. *Figs. made using Biorender.*

In this perspective, we first present an overview of numerous stresses that are connected to polyploidy, and the routes by which such stresses generate whole genome doubling. Subsequently, we discuss both the advantageous and detrimental consequences of polyploidy on genomes, cells, and tissues when it is known. Finally, we discuss areas for future focus.

## 1. Causes: A Wide Variety of Stresses Trigger Polyploidy

Recently, there has been renewed interest in polyploidy as a generalized response to a myriad of stresses (11, 12). In this section, we survey diverse stresses that trigger polyploidy. For a selected subset of common stresses, we discuss examples in diverse organisms and explain how the stress is thought to trigger polyploidy.

### 1.1. Temperature.

Temperature change has the potential to promote polyploidy across species. At extreme temperatures, particularly in high heat, the fidelity of both mitotic and meiotic cell division is often impaired in a manner that allows genome doubling without subsequent division. While high heat is more frequently discussed, cold can also cause instability of machinery responsible for chromosome segregation, namely centrosomes and spindles, as well as instability of cytoskeletal components which enable cytokinesis via membrane contraction or new cell wall deposition (Fig. 1*A*) (13, 14).

Heat stress-linked somatic polyploidy has already been recognized for its relevance in human health. Physiologically, temperature stress is a significant factor in tumor microenvironments. Tumor temperature may be different than surrounding tissue due to differences in metabolism, vascularization, and inflammation (15). Exogenous heat application has also been explored as a combinatorial treatment to increase efficacy of radiation and chemotherapeutics (16, 17). Across multiple human cancer cell lines, heat promoted endopolyploidization by perturbing the formation and orientation of centrosomes or deteriorating the cytoskeletal contractile ring required for cytokinesis (17, 18). The resulting polyploid cancer cells exhibited genome instability as well as altered sensitivity to certain classes of chemotherapeutics, which can help guide future targeted treatment plans (17, 18).

Temperature stress-induced polyploidy is also pertinent in nonhuman animals and plants, in which it appears to increase tolerance to environmental temperature variation (19–23). This is highlighted by recent observations such as in insects, where the frequency of endopolyploidy is correlated to seasonal temperature fluctuations (22). In plants in particular, the effect of temperature on meiotic cell divisions has been extensively studied. In the model plant *Arabidopsis thaliana* (hereafter referred to as Arabidopsis) and multiple species within the plant *Populus* genus (poplars, aspens, and cottonwoods), heat was shown to disrupt microtubule arrays required for meiotic spindles and cytokinesis in pollen (24–26). Similar observations were made in fish eggs, where heat shock impaired centrosome and spindle formation (27). Acute cold stress was also able to interfere with the radial microtubule arrays guiding cell wall deposition during meiosis in multiple *Brassica* plant species and Arabidopsis pollen, thereby preventing cytokinesis (28, 29). In this era of concern regarding rapid climate change, temperature stress-induced polyploidy is increasingly relevant.

### 1.2. Pharmacological Interventions.

A variety of pharmacological treatments are linked with changes in ploidy (Fig. 1*A*). Including treatments for fungal infections, chemotherapeutics, and plant herbicides, these drugs interfere with vital cellular processes in order to impede cell growth.

For instance, azole drugs are commonly used to treat mucosal and invasive human fungal infections, as well as in agriculture to treat plant fungal pathogens (30, 31). Azoles block ergosterol biosynthesis and cause cell membrane stress. In some cells this membrane stress leads to an uncoupling of cytokinesis from DNA replication, resulting in polyploidization across diverse fungal pathogens in vitro and in mouse models (32–36). In some pathogenic yeast species such as *Candida albicans* and *Cryptococcus neoformans*, azole resistant aneuploid progeny (with gains and losses of individual chromosomes or chromosome segments) frequently arise from these polyploid intermediary cells (34, 37). Resistant isolates often carried increased copy number of azole resistance-associated genes, suggesting that ploidy-related changes in gene copy number and dosage can enable pathogens to evade targeted therapy.

Similarly, many antimitotic chemotherapeutics target tumor cells by destabilizing or blocking formation of the mitotic spindle, causing genome duplication by inhibiting chromosome segregation. Indeed, many tumors in which antimitotic chemotherapy is applied exhibit polyploidy (33). Applications of similar antimitotic drugs which destabilize or block microtubule polymerization have been widely used across numerous plant species both as herbicides and inducers of polyploidy (38, 39). This includes drugs such as colchicine, oryzalin, trifluralin, and propyzamide, which have been extensively used to generate organismal polyploid plants through application to somatic stem cell precursors of germline tissue (38). Whether plants, fungi, or cancer cells are the target of pharmacological treatment, it is apparent that polyploidization is enriched among organisms which survive direct perturbations to cellular mitotic processes.

### 1.3. Genotoxicity.

Many genotoxic or DNA damaging stress conditions are linked to increases in cellular ploidy across diverse species (Fig. 1*B*). These similar trends are attributed to the intimate connection between genome integrity and cell cycle regulation, as well as the deep evolutionary conservation or convergence of cell cycle regulatory mechanisms between species. Many components involved in the pathways for sensing DNA damage and initiating a response are conserved between plants and animals, either via direct molecular homologs (e.g., ATM, ATR, WEE1 in both plants and animals) or nonhomologous proteins of similar functional roles (e.g., p53 and p21 in animals versus SOG1, KRP, and SIM/SMR in plants) (40, 41). Repeatedly, these players appear linked to polyploidy and the DNA damage response across numerous species.

For instance, DNA replication stress in human and/or mouse macrophages, osteosarcoma cell lines, and mammary glands can induce the ATR-dependent DNA damage response pathway (42–44). Activation of this pathway stalls the cell cycle, precipitating downstream activity of p53, p21, and/or WEE1 which eventually leads to polyploidization via mitotic bypass (43, 44). DNA damage is also known to induce polyploidization in primary human keratinocytes expressing a high-risk human papillomavirus (HPV-16 E7), a process important in the understanding of disease progression (45). In the plant model Arabidopsis, both chemically induced double-stranded DNA breaks and ionizing radiation-induced DNA damage promote endopolyploidy in a manner dependent upon ATM/ATR-SOG1 signaling (46, 47). In the more rapidly dividing plant stem cell populations, however, double-stranded breaks instead induce ATM/ATR mediated programmed cell death (48). Interestingly, this trend in which differentiated or endocycling cells may evade DNA damage-induced cell death, whereas stem or actively dividing cells do not, is mirrored in animals. For example, endocycling *Drosophila melanogaster* (Drosophila) cells from a variety of tissue types repress the programmed cell death response downstream of the ATM-mediated DNA damage response to ionizing radiation (49–52). In endocycling cells, this apoptotic outcome is avoided by posttranscriptional repression of p53 and silencing of downstream proapoptotic genes (52). If such polyploid cells regain mitotic potential following DNA damage, this suggests that endopolyploidy has the potential to perpetuate damage and genome instability as seen in polyploid cancer cells (49, 53). Yet if both apoptosis and cell division remain blocked, endopolyploidy allows tissue function to be maintained in the face of genotoxic conditions. In plants, endopolyploidy is associated with greater growth in geographic areas with higher solar UV indices, while in humans and mice, endopolyploidy helps maintain normal development of skin cells exposed to UV and function of mammary gland cells subject to replicative stress (44, 54, 55). UV radiation and other genotoxic conditions are common concerns in both agricultural and human health contexts, highlighting the importance of endopolyploidy in DNA damage mitigation across disparate fields of study.

### 1.4. Nutrient Composition and Abundance.

Availability of nutrients is an essential requirement for organismal growth, enabling metabolic processes which generate energy stores and basic components for new structures. As nutrient concentration and composition varies, polyploidy in plants and animals has been linked to survival, nutrient use efficiency, and modulation of growth (56–59). Likewise, frequency of endopolyploidy appears responsive to both the quantity of nutrients available, whether scarcity or excess, as well as the type of nutrient present. For instance, starvation treatment of nematodes has been used for decades as a means to induce polyploidization (60, 61). In some mosquito populations, blood feeding can increase endopolyploidy of midgut enterocytes, depending on the source of blood (58). In livers of mice subjected to intermittent fasting and experimental blockage of cell proliferation, endopolyploidization increases in frequency relative to nonfasting mice. Similarly, in human aortic endothelial tissue, high levels of glucose increases the accumulation of polyploid cells (62). The mechanistic connections between dietary stress and polyploidy vary widely in their current depth of understanding in these instances. However, in many cases it is suggested that changes in mitochondrial activity accompanying shifts in diet may modulate reactive oxygen species (ROS) accumulation and thereby endopolyploidy via mechanisms discussed further in Section 1.7 (Fig. 1*C*) (58, 62).

Compared to the digestive processes of animals or insects, in plants the main limiting nutrient to growth is often soil nitrogen content. For instance, frequency of endopolyploidy in Arabidopsis is lower under nitrogen scarcity and higher when nitrogen is abundant in the form of nitrate (57). These differences in endopolyploidy were found to be regulated by proteins which directly sense nitrogen concentration and modulate cyclin-dependent kinases involved in cell cycle progression (57). Later studies found that ammonium more greatly promoted endopolyploidy than nitrate if provided as the main source of nitrogen, likely because of ammonium’s ability to increase ROS accumulation and trigger an oxidative stress response leading to endopolyploidy (63). The mechanistic link between polyploidy, nutrient composition changes, and oxidative stress is still not fully understood.

### 1.5. Infections.

Just as pharmacological treatments can cause target pathogens to become polyploid, infectious agents themselves are also associated with polyploidization of their host tissue. As the pathogen seeks to accumulate nutrients from the host and replicate itself, it often modulates host cell cycling.

Many parasitic infections from insects, nematodes, or bacteria in the leaves or roots of plants result in the formation of a gall, an abnormal outgrowth which encapsulates and feeds the parasite. The cells composing galls are more frequently polyploid than the surrounding tissue, with endopolyploidy contributing to growth of established galls rather than gall initiation (64, 65). While mechanisms linking gall development to endopolyploidy have yet to be entirely elucidated and vary depending on the species involved, infection of Arabidopsis by protist pathogens or nematodes triggers altered expression of numerous regulators of the G2-M cell cycle checkpoint which are known to influence frequency of endopolyploidization (Fig. 1*D*) (65, 66). When endopolyploidy is limited in the host tissue, growth of the gall and parasite is also limited, suggesting that endopolyploidy supports the growth and metabolic demands imposed on infected tissue (67). Polyploid cells may also support parasitic proliferation in mouse liver hepatocytes, where it has been shown that the malaria parasite *Plasmodium* preferentially infects polyploid cells (68). However, it is currently unknown whether this occurs due to parasite preference, differences in polyploid cell susceptibility, or postinfection modulation in host cell cycling. Many viruses are also known to trigger polyploidy in human hosts, both in carcinogenic infections such as human papillomavirus (HPV)-associated tumors and noncarcinogenic infections such as in the kidneys of human immunodeficiency virus (HIV) patients (45, 69). Although mechanisms linking viral activity to polyploidy vary between host tissue type, they often involve bypass of G2-M cell cycle checkpoints or inhibition of cytokinesis (Fig. 1*D*) (69).

### 1.6. Cell and Tissue Loss.

In multicellular tissues, acute mechanical injury can lead to loss of cells from a population and such cell loss decreases tissue mass. If this cell loss can be sensed, surviving cells can regenerate the lost tissue via increased cell cycling. While cell division is one mechanism to generate new cells, underlying gene expression in the injured tissue may suppress the mitotic phase of the cell cycle. In these cases, cell loss triggers cell cycle reentry from a quiescent state, resulting in endocycles that duplicate the genome (Fig. 1*B*). This polyploid cell cycle activity can regenerate the lost cellular mass. In this form of polyploid regeneration, the stress-induced cell loss is compensated for by enlarging the genome, and concurrently the size, of those cells that survive the stress.

There are multiple examples where stress-induced cell loss promotes polyploidy in the surviving cell population (4, 10, 46, 55, 70–76). Endopolyploidy has proven to be a contributor to the restoration of tissue mass following injury in human and additional vertebrate model systems of the liver, heart, lung, bladder, and kidneys, both in combination with cell proliferation and as a compensatory mechanism when cell proliferation is blocked (10, 71–75). In the Drosophila hindgut, ovarian follicle, and abdominal epithelium, endopolyploidy contributes to tissue regeneration upon acute cell loss (76). Less has been reported in plants regarding the relation between acute injury and endopolyploidy as a regenerative process in surrounding surviving cells. Perhaps this is because, as opposed to animals for which repair and regeneration of existing organs is vital, plants maintain meristematic niches from which additional organs can continuously arise throughout their lifespan. Even so, in some cases endopolyploidy is also found to occur in response to cell loss. In response to UV-B radiation or mechanical removal of tissue designed to mimic herbivory in Arabidopsis, it was found that induction of endopolyploidy contributes to the plant’s ability to compensate and regrow from damage (55, 77–79). Induction of endopolyploidization following loss of cells is linked to a variety of signaling pathways with the end result of altered expression of cell cycle regulatory factors. This includes hormonal signaling in response to damage, UV-responsive regulatory proteins, and mechanisms relating to secondary genotoxicity and oxidative stress as discussed in Sections 1.3 and 1.7 (78, 79). The repeated emergence of endopolyploidy in tissue regeneration demonstrates its importance in maintaining organ size and function in multicellular organisms.

### 1.7. Reactive Oxygen Species.

While there are numerous stress-associated triggers of endopolyploidy, the accumulation of reactive oxygen species (ROS) may be a common factor among many of the distinct scenarios discussed thus far. ROS can result from perturbed mitochondrial function, though bursts of ROS are also initiated as signaling molecules in response to a variety of stress stimuli (80–82). Oxidative stress from ROS accumulation can occur under conditions such as cytotoxic, genotoxic, and herbicidal drug treatments (46, 83); disease microenvironments found in the liver, heart, and cancer (84–87); as well as abiotic environmental conditions of extreme temperature (88, 89), water scarcity (90–92), salinity (93, 94), and nutrient abundance or compositional changes (58, 62, 95). While these appear to be vastly different stimuli, every condition listed above is associated with oxidative stress-induced polyploidy.

Oxidative stress directly increases DNA damage and causes mitotic arrest via the DNA damage checkpoint (96). Yet in numerous cases, this mitotic arrest still allows for continued rounds of DNA replication leading to endopolyploid cells (Fig. 1*B*) (96). For instance, in liver hepatocytes of mouse models for nonalcoholic fatty liver disease, high ROS oxidative stress triggers a mitotic DNA damage checkpoint and results in increased somatic polyploidization relative to nondiseased individuals (85). Similarly, in postnatal mouse cardiomyocytes and human aortic endothelial cells, increased ROS accumulation, DNA damage response activation, and arrest of cell proliferation is strongly suggested as the causative factor behind increased frequency of polyploidization (62, 86). Frequency of endopolyploidy decreases accordingly when ROS accumulation is reduced in human and mouse cardiomyocytes via treatment with antioxidants, up-regulation of gene products which decrease ROS, or lessening energetic demands such that mitochondrial respiratory rate is reduced (62, 84, 86). In these liver and heart contexts, levels of ROS accumulation and the resulting polyploidization are associated with worsened disease treatment outcomes and lessened tissue regeneration via cell proliferation, indicating a compelling therapeutic target (62, 84–86).

Oxidative stress is also likely to impact polyploidy in plant systems, although experimental evidence is less direct. Across a variety of plant species, polyploidy is often associated with increased tolerance of conditions which promote oxidative stress (11, 63, 90, 91). In Arabidopsis, oxidative stress has been indirectly linked to polyploidization in association with conditions such as drought, ammonium application, and herbivory, all of which are known to increase frequency of endopolyploidy (11, 63, 77, 91). Furthermore, endopolyploidy in plants can result directly from DNA damage response pathways known to be activated by oxidative stress, as discussed above in animal species, including when oxidative damage is a secondary result from a distinct primary stress (46, 47). The specific signaling components involved in DNA damage response are widely conserved and have been directly linked to polyploidization across plants, mammals, and fish, as was discussed in Section 1.3 (40, 46, 47, 85, 97). Thus, it appears plausible that a large degree of polyploidization across unique stress conditions and evolutionarily distant species may be attributed to induction of the DNA damage response pathway by oxidative stress and associated oxidative DNA damage.

### 1.8. Conclusion: Stress Generates Polyploidy.

From the preceding sections, it is clear that there are a finite number of pathways for a cell to become polyploid, upon which diverse stresses converge (Fig. 1). Future work will benefit from categorizing stress-induced polyploidy into these pathways. In some cases, polyploidy is an indirect effect of stress that blocks the cell cycle, and in other cases polyploidy is a programmed response by cells intervening in their own cell cycles to deal with a stress. In the next section we discuss the consequences of polyploidy.

## 2. Consequences: The Impact of Stress-Induced Polyploidy

The above survey introduces many ways that acute stress can lead to polyploidy. In this section, we discuss how the aforementioned stress-induced polyploidy impacts the biology of genomes, cells, and tissues. We frame the discussion around potential beneficial and detrimental impacts of stress-induced polyploidy, where there is evidence. For cases where the role of stress-induced polyploidy is unclear, we suggest that these are areas for future investigation, so that the field can more fully understand the role of polyploidy in a given context.

### 2.1. Genomes.

By definition, polyploidy profoundly impacts the genome, creating one or many additional copies of the entire genome which can have positive and negative consequences on the genome itself. We refer the reader to previous reviews on these topics (2, 3, 98), while here we highlight how stress-induced polyploidy further impacts the genome.

Genome duplication has great potential to enable further genomic alterations through both tolerating and causing instability. Tolerance of genotoxic conditions and genomic instability is facilitated by extra copies of the genome which buffer against various insults and alterations to the DNA. Although not formed in response to stress, developmentally endopolyploid Drosophila ovarian follicle cells are highly tolerant of genotoxic stress, suggesting endopolyploidy is a mechanism by which some cells may evade death or senescence triggered by the DNA damage response as was discussed in Section 1.3 (49, 50). Likewise, reports suggest that heat-induced polyploid cancer cells may be more resistant to genotoxic treatments, but still susceptible to antimitotic agents (17). To overcome this resistance, combinatorial targeting of processes required for polyploid cancer cell viability, such as mitotic kinesin activity required for chromosome segregation, may be necessary to select against polyploid cancer cells with resistance to genotoxic chemotherapeutics (99, 100).

In addition to increased tolerance of genomic changes, polyploidy promotes genomic alterations by decreasing the faithful propagation of the genome over time as cells divide. Reentry of endocycling Drosophila cells into mitosis introduces genomic instability and aneuploidy, resembling the instability in division of polyploid cancer cells (49, 53). Likewise, in heat-induced polyploid cancer cells, chromosomal missegregation is increased in comparison with diploid progenitors (18). Several mechanisms drive genome instability in polyploid cells, including 1) extra centrosomes formed when DNA is duplicated, which generate multipolar spindles leading to chromosome missegregation; 2) under and over replication of the genome during S phase; and 3) disruption of chromatin architecture due to inappropriate scaling of the CTCF protein that maintains boundaries between chromatin domains [for a review, see reference (2)].

**Fig. 2.**
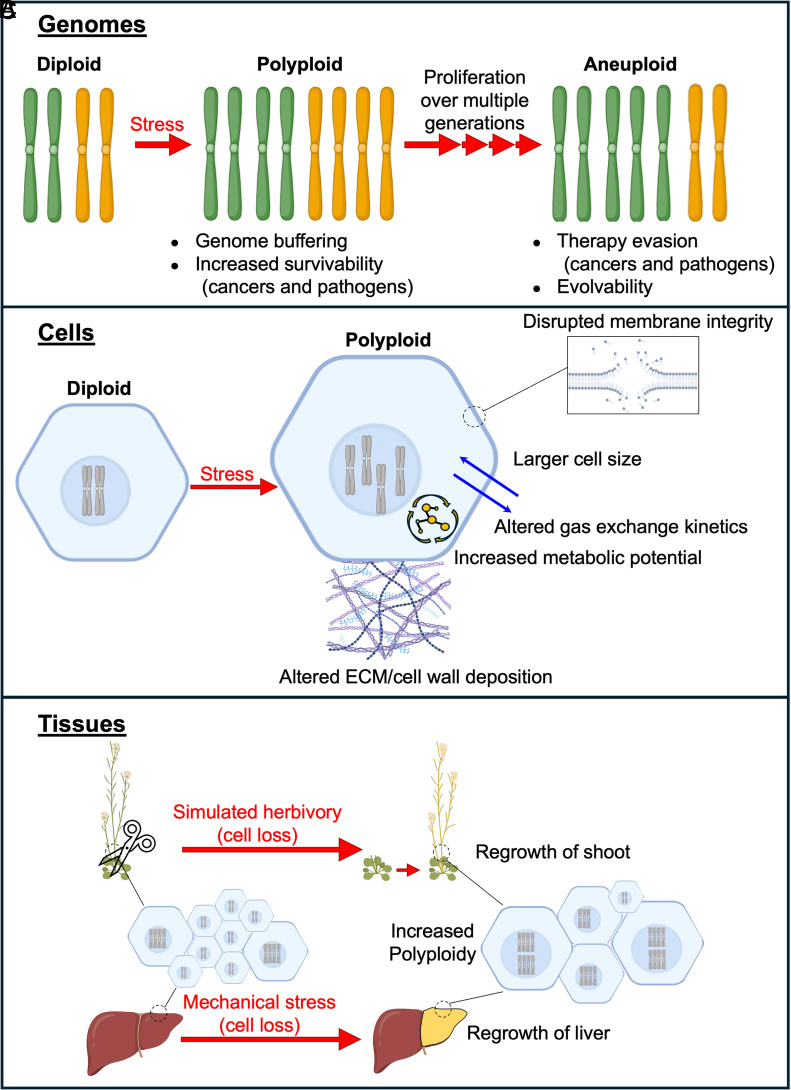
Consequences of stress-induced polyploidy. (*A*) Upon stress-induced polyploidy, many cells ultimately become aneuploid and exhibit differences in numbers of specific chromosomes. Consequences of polyploidy and subsequent aneuploidy on the genome are listed. (*B*) Impacts of polyploidy on the cell. (*C*) Consequences of polyploidy on tissues. *Upper*—Upon tissue removal, regrown plant tissue displays increased frequency of polyploid cells. *Lower*—Upon tissue loss such as following mechanical stress in the vertebrate liver, tissue regeneration can occur (denoted by coloration change). This regeneration is accompanied by the production of polyploid cells. *Figs. made using Biorender.*

Genome instability associated with polyploidy promotes changes conducive to evolvability. In many systems, polyploid cells undergo frequent chromosome missegregation resulting in a heterogenous population of aneuploid cells, and often a reduction in ploidy (Fig. 2*A*) (95, 101–103). This genomic heterogeneity can rapidly alter cellular phenotypes and provide adaptation to novel environments where aneuploidy is beneficial (37, 104, 105). Experimental and theoretical work indicates that reduction in ploidy from a polyploid fungal cell does not occur one chromosome at a time, but likely through a concerted mechanism where loss of one or more chromosomes predispose cells to lose others (102, 103). Polyploidy is associated with additional structural genome changes, including increased chromosome translocations and aberrations, recombination involving transposable elements, and transposon abundance (104, 106, 107). Ultimately, polyploidy seeds further genomic changes which can be acted upon by evolutionary selective pressures.

Another example of the impact of polyploidy on further genome evolution comes from long-term evolution experiments beginning with a diploid *Saccharomyces cerevisiae* (budding yeast) strain that forms multicellular clusters. In these studies, polyploid populations rapidly emerged following selection for enlarged cluster size (108). Polyploidization caused the cells to become larger and longer, both of which contributed to the formation of macroscopic cell clusters. Although tetraploid yeast genomes are inherently unstable, tetraploids were maintained as long as size selection was maintained. Interestingly, some of the evolving tetraploid lines became stable aneuploids which was associated with even larger cell size, cell length, and cluster size. Overall, an emerging pattern is that genome instability from polyploidy allows for further genomic innovation.

### 2.2. Cells.

In both normal and stress conditions, polyploidy alters the properties of the cell, most notably by generating cells of increased size with decreased surface area to volume ratio (109). This altered geometry of the cell impacts numerous processes (3) and can have both positive and negative consequences to the function and survival of polyploid cells due to changes in structural components of the cell (Fig. 2*B*).

Across multiple systems, cellular capacity to withstand pressure is altered in polyploid cells due to differences in cell size as well as cell membrane or wall composition. Large polyploid *Saccharomyces cerevisiae* cells were found to be more sensitive to turgor pressure, demonstrating increased viability when pressure was lowered by increasing osmolarity of growth medium or by using mutant strains which grow to smaller average cell sizes (110). These differences were linked to decreased expression of ergosterol biosynthesis genes in polyploid cells, which may impact membrane fluidity (110). In response to acute mechanical heart injury and senescence, polyploid heart cells demonstrated greater expression of proteins responsible for extracellular matrix deposition than their diploid counterparts (62, 111). This increased deposition of matrix components, such as matrix metallopeptidases, proline 4-hydroxylase α(II), collagens, and fibronectin, may be compensatory for lower structural stability in polyploid cells. This is likely the case in plants as well, wherein polyploid Arabidopsis demonstrated greater mechanical weakness (112). Yet perhaps as a compensatory measure, Arabidopsis polyploids also demonstrated increased enrichment of gene expression related to cell wall biosynthesis and associated processes (113, 114).

Polyploidy also impacts the biosynthetic capabilities of a cell, partly attributed to increased quantity of DNA template or organelles available for metabolic processes. In plant cells hosting parasitic or symbiotic interactions with a variety of fungi, insects, and other biotrophs, it is likely that endopolyploidy in host plant tissue helps accommodate increased metabolic requirements (115–117). Endopolyploidy in normal development of specialized cell types, such as plant trichomes or Drosophila nurse cells, is thought to support increased metabolic activity as discussed in prior reviews (3, 12). However, this increased cellular metabolism may come at the cost of increased ROS production. In human aortic endothelial tissue under high glucose conditions, the increased cellular respiration of polyploid cells is likely an additional source of oxidative stress in vascular disease (62). Alterations to the cell surface area and volume could also play a role in changes to the biosynthetic capabilities of polyploid cells. In the mosquito, it is proposed that blood feeding-induced endopolyploid cells may aid absorption by enlarging gut epithelial cells, increasing total surface area and enabling rapid processing of nutrients as they become available (58). Yet while enlargement of cells increases total surface area, it lowers the surface area to volume ratio. In metazoans such as **Xenopus*,* polyploid embryos consumed oxygen at a slower rate due to the lessened energetic demands required to maintain membrane potential in large polyploid cells with a lower surface area to volume ratio (118). Polyploidy in plants also alters gas diffusion, albeit for different reasons. In the mesophyll cells of vascular plant leaves, gas diffusion is limited by the lower cellular surface area to volume ratio and decreased packing density of larger polyploid cells (119). By hindering passive gas exchange, these limitations likely limit resulting photosynthetic rates (119).

It is clear that following an acute stress, newly formed polyploid cells are unique from their diploid counterparts. Further study of these cellular differences will help us to better understand the unique polyploid state in homeostasis and disease.

### 2.3. Tissues and Organs.

At the tissue level, stress induced polyploidy can be beneficial in diverse contexts. Much of the work on polyploidy at the tissue level pertains to tissue regeneration. After a mechanical injury, the ability of a tissue to restore lost cellular mass by increasing the ploidy and size of the remaining cells can be an effective form of regeneration, especially in the absence of stem or other highly proliferative cells. A classic example of tissue mass restoration by polyploidy is the liver (Fig. 2*C*) (120–122). A broadly used experimental regeneration model in rodents involves a partial hepatectomy, which removes specific lobes of the liver, or other stress treatments causing injury and cell loss. While this surgical injury does not lead to regrowth of the resected lobes, the remaining lobes enlarge to regenerate lost liver mass, and this occurs by increasing the ploidy of the hepatocytes in these regenerative lobes (122, 123). Even in mice subjected to intermittent fasting, polyploidization can maintain liver function if cell division is experimentally blocked (59). Additional examples of such productive regeneration by polyploidy include apoptosis-induced endoreduplication in the Drosophila hindgut pylorus (76, 124) and zebrafish epicardium (75), or by a combination of endoreduplication and cell fusion in the Drosophila abdominal epithelium (4, 125, 126). An advantage of regeneration through polyploidy/cell enlargement is that, unlike regeneration by cell division, cell contacts can be retained to maintain tissue integrity. Such altered tissue makeup in wounds may also facilitate more accurate healing, as human dermal wounds that healed well were found to have a higher percentage of polyploid cells than defectively healed wounds (127). In Arabidopsis plants subjected to mechanical damage mimicking herbivory, increased frequency of endopolyploidy was found to aid regrowth and mitigate detrimental effects on fitness caused by tissue loss (Fig. 2*C*) (77, 79). Similarly, polyploidy was found to improve tolerance of plants to UV or other genotoxic conditions which can cause cell loss (46, 55, 78). These examples highlight the increasingly recognized role of polyploidy as a powerful means to enable regeneration and maintenance of genome number in the face of environmental insults (128).

While endopolyploidization is evidently a useful means of tissue regeneration in the short-term, once present, polyploid cells may limit future proliferative capacity and alter long-term function in those same tissues. Especially in plant systems, once cells have committed to endopolyploidization they are almost never observed to divide (129). In rodent and fish heart models, polyploidy also appears to prevent proliferative restoration of cardiomyocytes upon cell loss (130–133). In the kidneys, initial polyploidization following acute injury aids organ function. However, in the long-term, polyploidy can drive senescence and functional decline (73, 134). A persistent alteration of kidney function or tissue architecture accompanies polyploidy upon both chronic kidney disease and HIV infection (69, 134, 135). Likewise, in the liver, regenerative polyploidy commonly maintains organ function following cell loss, yet the resulting polyploid hepatocytes ultimately grow slower and demonstrate a higher potential for cellular senescence (136–138). Intriguingly, these polyploid hepatocytes are also reported to be preferentially infected by the malaria parasite *Plasmodium*, although the reasons and functional consequences of this remain largely unknown (68). This pattern of parasitic enrichment within polyploid cells or tissues is reminiscent of gall formation in plants. Following infection by bacterial, viral, insect, or other parasites, gall formation causes altered tissue organization and function which feeds and shelters the parasite. Polyploidization is often enriched closer to the parasite, and supports the formation of the gall (64, 66). Polyploidization is therefore likely to be a limiting factor in proliferative expansion of tissue across a variety of species, at times severely altering tissue architecture and function.

From the above studies, it is clear that the sudden production of many polyploid cells upon an acute stress has the ability to both restore as well as disrupt tissue architecture and function. Future research to distinguish between these two scenarios will be essential, and could enable the therapeutic and resilient potential of polyploid cells to be harnessed in disciplines ranging from medicine to agricultural crop production.

## 3. Conclusion and Future Outlook

In this perspective, we have highlighted diverse stresses that generate polyploidy. Within this diversity, it is intriguing that similar stresses can generate polyploidy in very different organisms and tissues, both in normal and diseased settings. The emerging theme is that each stress impedes cell division whether through inhibiting M phase of the cell cycle, causing DNA damage and triggering a checkpoint, affecting the spindle and chromosome segregation, or preventing cytokinesis, which all cause the cell to double its DNA content.

Further, regardless of organism or stress, there are common impacts of stress-induced ploidy changes on the very different biological scales of genomes, cells, tissues, and organisms. The consequences of the ploidy change can be positive, negative, or neutral in withstanding the stress and in the ongoing life of the organism. Comparison across organisms has been instrumental in uncovering the overarching principles of polyploidy from the specifics of the organism and situation. We suggest that further integration of data from different polyploid cells and organisms is an important avenue for future advances in our understanding of how polyploidy creates multiscale effects from genomes to cells to tissues and organisms. Yet, context-specific differences remain, and these differences are important in understanding the impact of a given stress on polyploidy in that setting.

A common misconception of polyploidy is that it is a rare event—yet the study of organisms under stress already demonstrates that polyploidy is very common and a major player in a myriad of life’s processes. This recognition of the prevalence and functional role of polyploidy is likely only to increase. While quite a bit is understood about how stresses cause polyploidy, less is known about the consequences. Future work should focus on the grand challenges of 1) further elucidating the mechanisms through which merely doubling (or multiplying) the DNA content changes the cell; 2) how these cellular effects propagate to the tissue and organism scales; and 3) how the consequences of polyploidy affect the organism’s resilience to stress.

## Data Availability

There are no data underlying this work.
